# Novel Strategies against Cancer: Dexibuprofen-Loaded Nanostructured Lipid Carriers

**DOI:** 10.3390/ijms231911310

**Published:** 2022-09-25

**Authors:** Vaikunthavasan Thiruchenthooran, Marta Świtalska, Lorena Bonilla, Marta Espina, Maria Luisa García, Joanna Wietrzyk, Elena Sánchez-López, Anna Gliszczyńska

**Affiliations:** 1 Department of Food Chemistry and Biocatalysis, Wrocław University of Environmental and Life Sciences, Norwida 25, 50-375 Wrocław, Poland; 2 Department of Experimental Onclogy, Ludwik Hirszfeld Institute of Immunology and Experimental Therapy, Polish Academy of Sciences, Weigla 12, 53-114 Wrocław, Poland; 3 Department of Pharmacy, Pharmaceutical Technology and Physical Chemistry, University of Barcelona, 08028 Barcelona, Spain; 4 Institute of Nanoscience and Nanotechnology (IN2UB), University of Barcelona, 08028 Barcelona, Spain; 5 Unit of Synthesis and Biomedical Applications of Peptides, IQAC-CSIC, 08034 Barcelona, Spain

**Keywords:** dexibuprofen, anticancer activity, nanostructured lipid carriers (NLCs), factorial design, particle size, zeta potential, cytotoxicity, drug delivery

## Abstract

The aim of this work was to design innovative nanostructured lipid carriers (NLCs) for the delivery of dexibuprofen (DXI) as an antiproliferative therapy against tumoral processes, and overcome its side effects. DXI-NLC samples were prepared with beeswax, Miglyol 812 and Tween 80 using high-pressure homogenization. A two-level factorial design 2^4^ was applied to optimize the formulation, and physicochemical properties such as particle size, zeta potential, polydispersity index and entrapment efficiency were measured. Optimized parameters of DXI-NLCs exhibited a mean particle size of 152.3 nm, a polydispersity index below 0.2, and high DXI entrapment efficiency (higher than 99%). Moreover, DXI-NLCs provided a prolonged drug release, slower than the free DXI. DXI-NLCs were stable for 2 months and their morphology revealed that they possess a spherical shape. In vitro cytotoxicity and anticancer potential studies were performed towards prostate (PC-3) and breast (MDA-MB-468) cancer cell lines. The highest activity of DXI-NLCs was observed towards breast cancer cells, which were effectively inhibited at 3.4 μM. Therefore, DXI-NLCs constitute a promising antiproliferative therapy that has proven to be especially effective against breast cancer.

## 1. Introduction

Cancerous diseases are the second leading cause of death worldwide, representing a serious health concern. According to the World Health Organization (WHO) report, by 2040 the number of cancer patients will increase by 47% compared to 2020, and will reach more than 28 million cases and 16 million deaths [1]. Hence, many research centers are looking for effective compounds in the fight against cancerous diseases. In 2020 and 2021, the Food and Drug Administration (FDA) approved 53 and 50 new drugs based on 40 and 36 new chemical entities (NCEs), respectively [2,3]. In 2020, 18 new anticancer drugs were released on the market, whereas in 2021 only 15 were found [4].

Developing and bringing a new drug to the market constitutes a tedious and expensive procedure that takes, on average, twelve to sixteen years and requires USD 2.558 billion [5]. The pharmacologist and Nobel laureate James Black used to say that “the most fruitful basis for the discovery of new drug is to start with an old drug”. Applying his principle, it is possible to bypass almost 40% of the overall cost of bringing a drug to market [6]. This is because drugs approved by regulatory agencies with known pharmacokinetics and safety profiles can be rapidly evaluated in phase II clinical trials where their novel application is confirmed. 

Turning to drugs that have already been recognized as safe by the FDA is being increasingly recognized as an attractive strategy to obtain effective treatments. Consequently, many efforts have been made for repurposing drugs that already exist by finding new targets, delivery methods and formulations.

In the area of tumoral processes, great hopes are placed on NSAIDs as effective antiproliferative agents, as these have exhibited the ability to reduce the incidence and mortality of many types of cancer in numerous epidemiological and animal studies [7]. It is suggested that anti-inflammatory drugs prevent malignant cell formation and tumor progression. They may also promote the antitumoral effects of chemotherapy and radiotherapy [8]. The first mention of the use of this group of drugs as anti-tumoral compounds appeared during the 1980s [9]. Harris and co-workers reported that their regular use for 5 to 9 years caused a 21% reduction in the incidence of breast cancer [10]. However, despite promising in vitro results and clinical trials assessing their anticancer activity, the practical application of these drugs is still impossible because of individual adverse effects, such as increased gastrointestinal ulceration arising after their long-term administration [11]. Therefore, in recent decades, many attempts have been made to find the effective solution for their delivery to overcome these side effects. 

One of the NSAID drugs with proved anticancer potential is ibuprofen (IBU), the mechanism of action of which has been extensively studied. Greenspan et al. suggested that IBU can effectively target both the Wnt/β-catenin and NF-κB pathways, indicating a novel mechanism of chemopreventive efficacy of NSAIDs [12]. Another research group published that this drug regulates expression and function in epithelial cells [13]. 

Dexibuprofen (DXI) is the dextrorotatory isomer of ibuprofen (IBU), being almost 160 times more active as an anti-inflammatory molecule and less toxic than its enantiomer *R* [14]. DXI is better tolerated in comparison with other NSAIDs, but still exhibits some side effects characteristic of this group of drugs. Therefore, encapsulation and methods of fabrication of the lipid nanostructures of IBU have been studied [15,16,17].

The aim of this work was to develop a controlled-release DXI delivery system based on lipid nanocarriers. Among them, nanostructured lipid carriers (NLCs) were selected due to their excellent pharmaceutical and drug delivery properties, in addition to their biocompatibility and biodegradability [18]. NLCs are able to encapsulate lipophilic drugs and are formed by mixing solid and liquid lipids with aqueous surfactant dispersion [19]. NLCs provide some advantages over traditional solid lipid nanoparticles (SLNs) and other colloidal lipid carriers. On one hand, both NLCs and SLNs are known to provide a prolonged drug release due to the presence of a solid lipid matrix [20]. However, SLNs possess a restricted stability due to the fact that they tend to expel the drug from the lipid matrix. Thus, NLCs overcome SLN problems due to the incorporation of a liquid lipid that offers higher drug loading capacity, as well as increased stability. Using NLCs, it would be possible to obtain a formulation providing prolonged-release DXI, and to produce the formulation at an industrial scale [21]. Therefore, during this work, the preparation, physicochemical characterization and therapeutic efficacy of DXI-loaded NLCs will be assessed.

## 2. Results and Discussion 

### 2.1. Preformulation Studies 

The selection of solid lipid used in the preparation of NLCs was made based on the literature data indicating the antibacterial properties of beeswax against Gram-positive bacteria and its anti-inflammatory activity, which could contribute to enhancing the antiproliferative properties of DXI [22,23].

**Figure 1:**
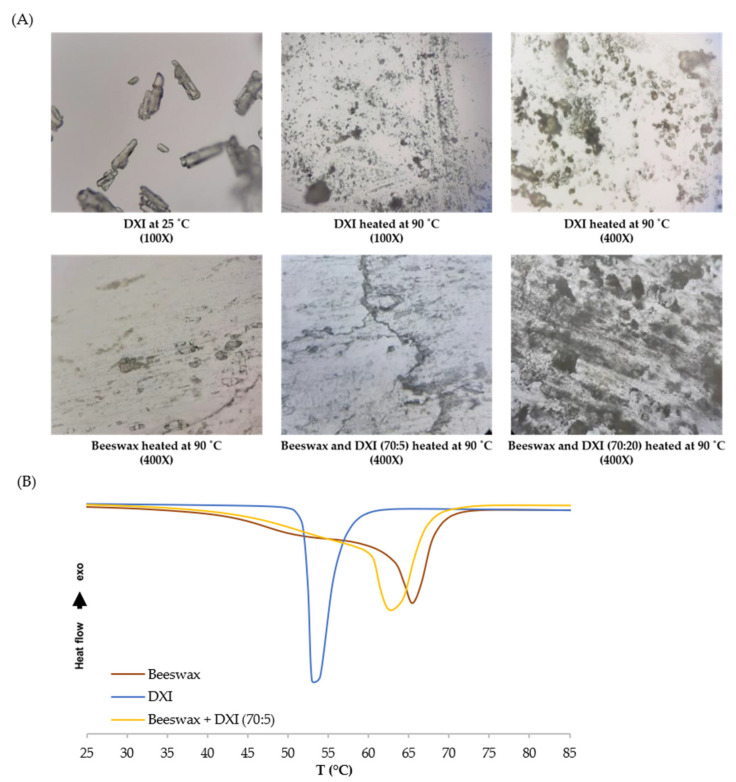
Optical microscopy images (**A**) and differential scanning calorimetry (**B**) of physical mixture of beeswax and DXI.

Knowledge about interactions between the solid lipid and the drug is crucial in the process of designing nanocarriers for further drug entrapment [24]. First, we examined the interactions between beeswax and DXI by preparing two different mixtures of beeswax:DXI in ratios 70:20 and 70:5 (%, *w*/*w*). Samples of DXI and beeswax, as well as their mixtures, were heated at 90 °C, cooled down to room temperature, and finally visualized under a light microscope. Under the microscope, a noncomplete mixture of DXI with solid lipid at a ratio of 70:20 was observed. The observable dark opaque areas denote the area of recrystallized DXI. In contrast, the 70:5 ratio had a reduced exposure of dark opaque DXI recrystallisation under light microscopy. Based on this observation, the ratio 70:5 of beeswax: DXI was selected and examined by differential scanning calorimetry (DSC). DSC constitutes an effective method to investigate the melting and crystallization behavior of lipid molecules and drugs. Thermograms were obtained by heating the samples (beeswax, DXI and beeswax:DXI) from 25 °C to 100 °C (Figure 1B). The data obtained indicate that the melting point of DXI was 53.6 °C, the melting point of beeswax was 65.94 °C, and the melting point of their mixture was 62.4 °C. 

This increase in the melting point of the mixture against the DXI melting point, broadening the endothermic curves, corresponds to the solubilization of the lipid compound with the drug. Using DSC studies, we confirmed microscopic results in order to ensure that DXI could be completely dissolved into the solid lipid [25]. Furthermore, we also observed a slight reduction in the heating enthalpy of beeswax (∆H 134.14 J/g) during its mixture with DXI (∆H 119.93 J/g). Applying heterogenic solid lipids with a higher melting point has the capacity to disorder the recrystallization of drugs, and during this study it is evidenced that beeswax prevents DXI from undergoing recrystallization, and could help improve the entrapment of DXI in the beeswax matrix during DXI-NLC formulation [24]. 

Afterwards, liquid lipids were selected by investigating castor oil and Miglyol 812. Castor oil is a natural oil with high viscosity. Its main component is a ricinoleic acid: glyceride [26]. Miglyol 812 is a mixture of triglycerides of saturated and unbranched C8 to C12 fatty acids, and is characterized by high chemical stability and low viscosity [27]. Both products are water-insoluble and non-ionic lipids with different HLB values (14 and 15.36, respectively). Miglyol 812 has been described to possess antiproliferative activity and it has been widely used as liquid lipid in NLCs with a broad range of antitumoral applications [28,29]. Castor oil is considered an important plant product in herbal medicine. It is known to penetrate well through human skin and eradicate tumors near the breast skin surface [30]. 

Tween 80, an oleate ester of sorbitol, was used as a surfactant. Tween is a water-soluble and non-ionic synthetic product with an HLB of 15. 

With the selected excipients, preformulations A and B were performed using the concentrations given in Table 1 and the high-pressure homogenizer (HPH). The HPH, in which tension is generated by high pressure, is the main method to produce lipid nanoparticles due to its feasibility for scale-up, short production time, uniform particle size reduction, and the avoidance of organic solvents [31]. Despite this, the number of cycles and the pressure applied should be optimized, since these are the critical parameters in HPH.

Physicochemical parameters of both formulations were evaluated. Based on the results summarized in Table 2, Miglyol 812 was selected for further studies instead of castor oil because it showed better properties in terms of polydispersity index (PDI, obtaining values below 0.2) and mean particle size (Z_ave_ around 100 nm), while in terms of entrapment efficiency (EE) and zeta potential (ZP), the results were similar in both cases, obtaining values higher than 95% and lower than −14 mV.

### 2.2. Optimization of Dexibuprofen–Nanostructured Lipid Carriers 

In order to obtain the final desired product with optimal physiochemical properties and maximal DXI encapsulation, the design of experiment (DoE) approach was used. The effects of the formulation variables (independent variables) on the response parameters (dependent variables) were evaluated. According to the matrix generated, 26 experiments (16 factorial points, 8 axial points, and 2 replicated center points) were performed (Table 3) [32]. 

**Figure 2:**
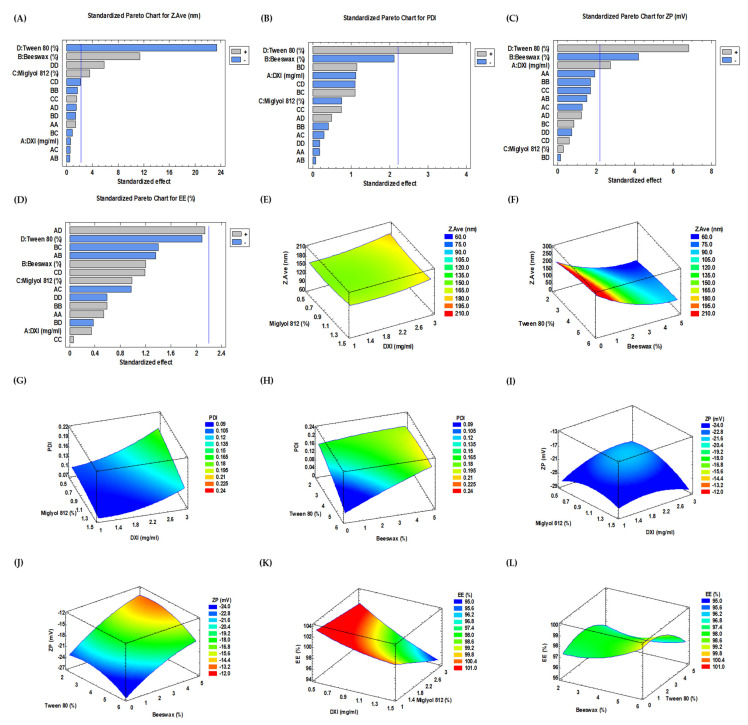
(**A**–**D**) Pareto chart shows positive and negative influence of dexibuprofen (DXI), Miglyol 812, Tween 80 and beeswax on the particle size (Z_ave_), polydispersity index (PDI), zeta potential (ZP) and encapsulation efficiency (EE). (**E**–**L**) Surface response plots denoting changes in Z_ave_, PDI, ZP and EE during different concentrations of DXI, Miglyol 812, Tween 80 and beeswax. (**A**) Pareto chart for Z_Ave_; (**B**) Pareto chart for PDI; (**C**) Pareto chart for ZP; (**D**) Pareto chart for EE. (**E**,**F**) Surface response plot for Z_Ave_; (**G**,**H**) surface response plot for PDI; (**I**,**J**) surface response plot for ZP; (**K**,**L**) surface response plot for EE. In the Pareto chart, a bar crossing the blue line is considered a significant effect.

The results obtained were analyzed and, as can be observed in Figure 2, on the Pareto’s chart of Z_ave_, it is clearly visible that the statistically significant variables corresponded to Tween 80^®^, beeswax and Miglyol (Figure 2). 

Regarding the mean particle size, site-specific NLCs aiming to deliver chemotherapeutic agents should have a diameter range of 50–300 nm for increased cellular uptake [33]. In our studies, we observed that the average size of the DXI-NLC strongly depended on the concentrations of Tween 80^®^ and beeswax, and was reduced with the increase in the surfactant (Figure 2A,F), whereas the opposite tendency was reported for beeswax. At concentrations of Tween 80^®^ lower than 2%, the average size of DXI-NLC decreases. On the other hand, it was observed that the concentrations of DXI do not influence this parameter in a significant manner (Figure 2A,E).

The PDI has an Important effect on the physical stability and uniformity of NLC. The values of this parameter should be as low as possible to ensure the long-term stability of the formulation. PDI values in the range between 0.1 and 0.25 show a narrow size distribution, while PDI values greater than 0.5 indicate a very broad distribution [34]. In the conditions studied, the concentrations of Tween 80^®^ and beeswax have a strong impact on the PDI (Figure 2B,H). Since the homogeneity of the formulation is a key issue, low PI is sought. Therefore, according to our data, the concentration of surfactant should be high while the solid lipid should be maintained as low as possible. In the case of DXI, a nonsignificant impact of its concentration was observed.

All obtained formulations had a negative surface charge ranging from –13.1 to –21.8 mV (Table 3), predicting a suitable short-term stability regardless of DXI concentration, and thus suggesting that DXI did not significantly alter the ZP of the formulation. This is in accordance with previous studies which point out that when beeswax is used the formulations possess anionic character [35]. These negatively charged NLCs have higher half-life during circulation and are less likely show toxicity because of their relatively low interaction with the mononuclear phagocytic system [36]. In addition, in the blood stream, the majority of plasma proteins are negatively charged, and cationic NLCs would bind to these proteins at a higher rate [37,38]. Taking all this into account and aiming for suitable short-term stability, highly negative ZP values should be obtained. Therefore, high beeswax amounts would be suitable since values around −20 mV can be obtained (Figure 2C,J). 

The concentrations of DXI, beeswax and surfactants had no significant impact on the encapsulation efficiency (EE) of DXI (Figure 2D). As it can be seen in Table 1, a large amount of DXI (EE > 99%) was incorporated in some DXI-NLC formulations, suggesting its preferential partition into the lipid matrix of the NLCs. From the surface response chart (Figure 2L), we can find that at a lower concentration of Tween 80^®^ and a higher concentration of beeswax, the EE parameter is higher (Figure 2L). 

The DoE approach allowed us to develop an optimized DXI-NLC. Based on the analyzed data, 10 mL samples were prepared using 600 mg of beeswax, 200 mg of Miglyol, 200 mg of Tween 80^®^, and 15 mg of DXI for their subsequent characterization (Table 4). Samples were prepared in triplicate to verify their reproducibility and stored under the same conditions. As shown in Table 4, predicted and experimental values were highly similar, except for the EE, which was higher than expected.

### 2.3. DXI-NLC Characterization Studies

**Figure 3:**
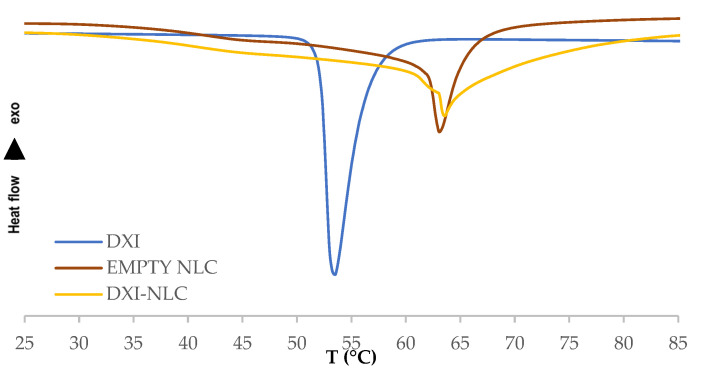
Differential scanning calorimetry (DSC) of DXI-NLC, DXI and empty NLC.

In order to study the thermal profile of DXI-NLC, DSC of dried DXI-NLC, as well as the individual compounds, was carried out. Figure 3 shows that DXI exhibits a melting point of 53.6 °C, while the melting point of the empty NLC is 63.03 °C. The DXI-NLC melting point is similar to the empty NLC (63.06 °C). These data confirm that DXI is in a disordered crystalline state without exhibiting its melting peak in DXI-NLC [39]. 

**Figure 4:**
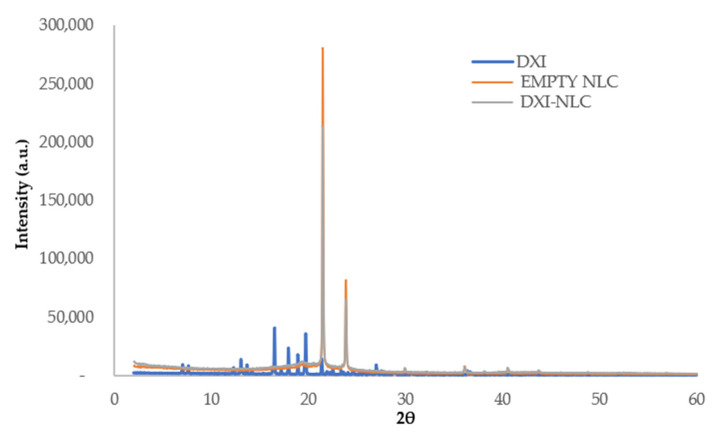
X-ray diffractogram pattern of DXI-NLC, DXI and empty NLC.

An X-ray diffractogram (XRD) was performed to characterize the DXI-NLC crystalline in its amorphous state (Figure 4). The XRD profiles of DXI present sharp peaks indicating crystalline structure, while the empty NLC shows two high diffraction peaks (2θ: 21.4 and 23.8) corresponding to orthorhombic subcell diffraction of waxes [40]. Interestingly, in DXI-NLC, the intensity of these two peaks slightly dropped. 

Beeswax has a heterogeneous chemical composition that makes it less crystalline, and the reduction in sharpness of these peaks in DXI-NLC shows the changes in the packing β-modification of beeswax [40]. The lack of peaks, corresponding to DXI not being visible in the drug-loaded particles, indicates that DXI is present in the dissolved state (molecular dispersion). Moreover, no peaks of DXI can be observed in DXI-NLC, thus indicating that DXI is encapsulated in the NLC matrix.

**Figure 5:**
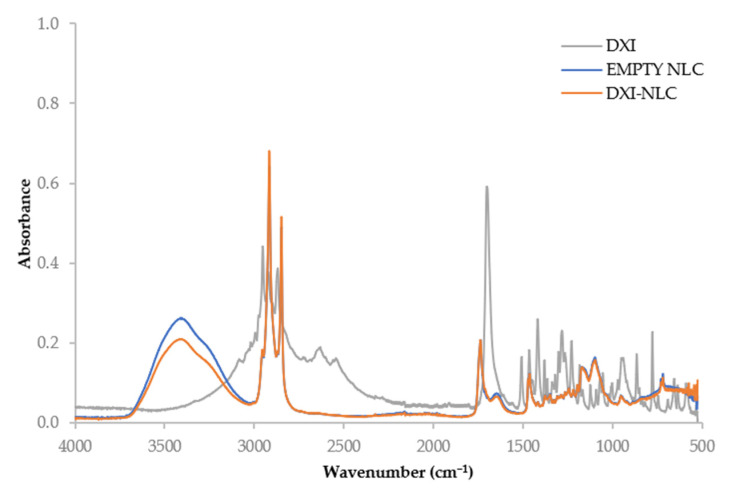
Fourier-transform infrared spectroscopy (FTIR) of DXI-NLC, DXI and empty NLC.

Fourier-transform infrared spectroscopy (FTIR) is a useful tool to confirm the analysis of bonds between molecules. Thus, FTIR was performed to identify the interaction between DXI and the lipid matrix. In Figure 5, it can be observed that there are no new covalent bonds formed between DXI and NLC. The main peak characteristic for DXI from the C=O bond-stretching vibration is presented at 1701 cm^−1^. Other small peaks from C-C bonds (1508 cm^−1^_,_ 1466 cm^−1^ and 1417 cm^−1^), C-O stretching (1282 cm^−1^) and O-H (778 cm^−1^) were also observed [41]. 

In the cases of the empty NLC and DXI-NLC, similar vibrations attributed to bonds appeared. There were several vibrations observed at the beeswax fingerprint region (719–1098 cm^−1^). The bond vibrations corresponded to the CH_2_ rocking (719 cm^−1^), C-H bending (1098 cm^−1^), and C=O stretching (1737 cm^−1^) of hydrocarbons, esters and free fatty acids in beeswax [42]. The major vibrations between 2848 and 2915 cm^−1^ were attributed to the symmetric and asymmetric CH_2_ stretching vibrations of hydrocarbon compounds present in beeswax, respectively. 

The notable change during DXI loading is shown in the reduction in the water molecular O-H bond-stretching vibration at 3411 cm^−1^. This is an indication of the dispersant being present in the interfacial or pocketed regions of beeswax, and being incompletely removed during DXI loading because of its hydrophobicity. There were no reportable additional bond vibrations observed between the empty NLC and DXI-NLC. 

**Figure 6:**
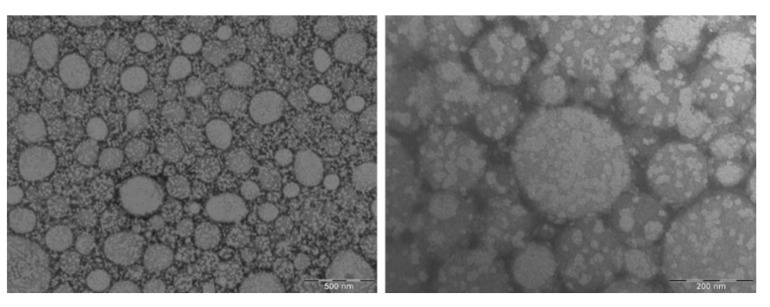
Transmission electron microscopic images of DXI-loaded nanostructured lipid carriers (DXI-NLC) with 500 nm and 200 nm scale.

The morphology of DXI-NLC was observed using transmission electron microscopy (TEM). The images presented in Figure 6 confirm that there was no aggregation between the nanoparticles. DXI-NLCs were spherical and uniform in shape, with smooth surfaces. This is relevant because the shape of the colloidal dispersion system is known to affect the delivery and response in medicinal applications, and spherically shaped formulations are known to have a better immune response [33]. Moreover, for the effective delivery of active molecules in the body, the size of nanoparticles is also largely responsible. NLC populations with Z_ave_ above 200 nm are known to compromise the human complement system, whereas Z_ave_ values below 10 nm are known to be excreted by the human kidney [33]. Therefore, the Z_ave_ measured using DLS was similar to that obtained with TEM, thus corroborating the results obtained where NLC measured less than 200 nm.

### 2.4. In Vitro Drug Release

**Figure 7:**
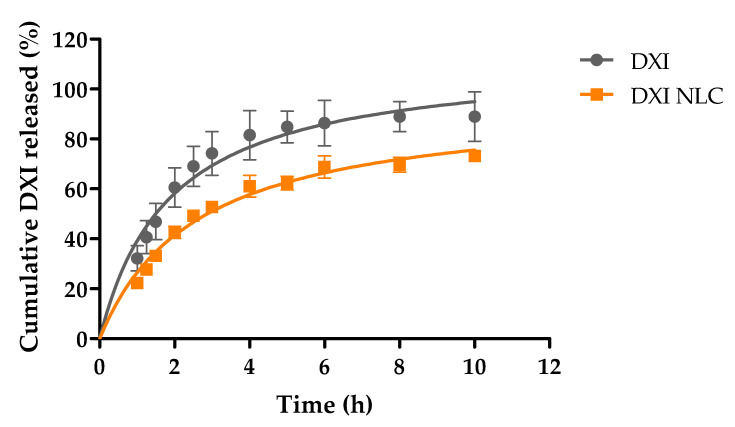
Released profile of free DXI and DXI-loaded nanostructured lipid carriers (DXI-NLC) after 10 h of incubation, adjusting the data to a hyperbola equation.

The release profiles of free DXI and DXI encapsulated in NLCs are shown in Figure 7. The release medium had been prepared at pH7 to contain sodium citrate and urea at concentrations of 1.74 M and 0.18 M, respectively, to ensure sink conditions throughout the experiment. During this study, the faster release kinetics of free DXI in comparison to DXI-NLC can be observed. After 10 h, the drug was almost fully released, whereas after this time only 73% of the initial amount of drug was released from the NLCs. Moreover, significant differences were obtained by comparing the release data one hour after release (*p* < 0.05). Before one hour, no significant differences between free DXI and DXI NLC were found. This could be due to the fact that, during the first hour, the DXI that was located on the NLCs’ surface was released in a similar manner to free DXI due to a burst effect [43,44]. After the first hour, the DXI that was encapsulated in the inner core started being released at a slower rate than the free DXI, causing significant differences. These two phases confirm that DXI-NLC possesses a kinetic profile characteristic of prolonged drug-release formulations [45].

Drug release is affected by many other factors, such as morphometric and morphological paraments, the type of surfactant, and preparation methodology [46]. In the literature, it is reported that a faster release occurs due to drug diffusion, and could not be caused by matrix degradation, as is the case in DXI-NLC [47]. However, this burst release is one of the limitations among SLN and NLC, as they tend to show an initial fast release [48]. To further understand how DXI is released from formulations, the results were fitted to the most common biopharmaceutical equations and the best fit corresponded to the hyperbola equation [49]. Comparatively, a higher equilibrium dissociation constant (K_d_), as is the case of DXI-NLC, reports a slower drug release from the NLC solid lipid matrix, thus confirming the slower DXI release from the particle matrix (Table 5) [39]. 

### 2.5. Short-Term Storage Stability 

Although NLCs are known to be more stable than SLNs, still one of the major limitations of NLCs is their restricted storage stability [20]. In order to address this issue, the optimized DXI-NLCs were stored at three different temperatures (4, 25 and 37 °C) for up to 60 days, and the resulting modifications in the physiochemical parameters were measured (Table 6). In addition, NLCs are limited to lipophilic drugs such as DXI, and are less suitable for hydrophilic drugs [20].

Although the HPH technique provides a uniform rigid NLC population, during storage, Z_ave_, PDI and ZP could be altered by multiple factors [43]. Analyzing the components of fabricated formulation, it can be pointed out that Tween 80^®^ is known to provide suitable storage stability against other surfactants [50,51]. However, waxes and glycerides are sometimes avoided for NLC preparation due to low encapsulation and drug expulsion [40]. In our case, beeswax at 30 days releases a low percentage of DXI, and this is lower at 4 than at 25 °C (2.35% and 3.25%, respectively). From the obtained results, initial and 60-day storage showed nonsignificant physicochemical modifications also denoting that DXI-NLC could be better stored at 4 °C than 25 °C in the long term. As explained by Freitas and Müller, to retain size, formulations should be stored at a low temperature between 2 and 8 °C [52]. However, the 4 °C temperature should be studied for long-term periods to confirm these data. Furthermore, storage at 37 °C causes a decrease in ZP values, probably due to alterations in the formulation, as has been previously reported in other studies [53].

### 2.6. Effect of Gamma Radiation 

Before assessing the antiproliferative activity of DXI-NLC, sterilization using gamma radiation was performed. Gamma radiation is performed at the recommended dose of 25KGy, which is known to kill microbial contaminants [54]. This sterilization method is preferable due to NLCs’ heat-sensitive properties, and methods using high temperatures such as ethylene oxide or autoclaving have been reported to cause significant adverse impacts on the physicochemical properties of NLCs [50].

**Figure 8:**
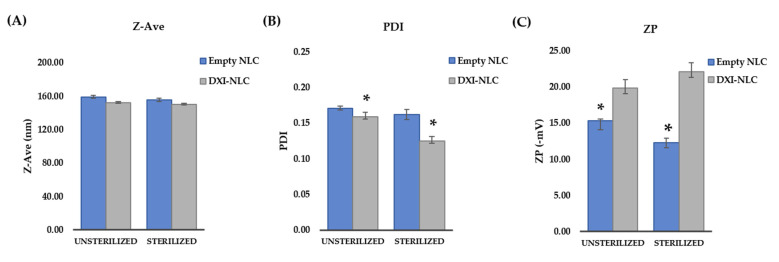
Effect of gamma sterilization on DXI-loaded nanostructured lipid carriers (NLC). (**A**): bar chart for sterilization effect on size (Z-Ave) of empty NLC and DXI-NLC; (**B**): bar chart for sterilization effect on polydispersity index (PDI) of empty NLC and DXI-NLC; (**C**): bar chart for sterilization effect on zeta potential (ZP) of empty NLC and DXI-NLC; * significant difference.

Data on the physicochemical parameters after gamma sterilization are shown in Figure 8. As can be observed, NLC Z_ave_ was not significantly affected by sterilization (Figure 8A). However, the ZP of both the empty NLC and DXI-NLC was affected significantly (*n* = 3, *t* statistics: 9.986, *p* value: 0.001), as was PDI in the case of DXI-NLC (*n* = 3, *t* statistics: 8.302, *p* value: 0.001) (Figure 8B,C). Despite this, ZP was still negative and PDI was below 0.2. DXI EE remained higher than 99%. Small changes in chemical structural parameters were found in previous NLC preparations, but did not show any impact on the medicinal properties. After sterilization, the empty NLC had a Z_ave_, PDI and ZP of 155.3 ± 3.21 nm, 0.16 ± 0.01, and −12.3 ± 0.64 mV, respectively, whereas the DXI-NLC properties were 150.3 ± 2.98 nm, 0.13, and −22.1 ± 0.75 mV, respectively. 

### 2.7. Cytotoxicity of Fabricated Formulation towards Selected Cancer Cell Lines

The antiproliferative activity of DXI and DXI-NLC towards prostate (PC-3) and breast (MDA-MB-468) cancer cell lines was evaluated after 24, 48 and 72 h. The results were obtained with an cellular viability assessment (SRB colorimetric assay). The data on in vitro anticancer activity are reported in Table 7, and are expressed as the IC_50_ concentration of the compound (in μM) that inhibits the proliferation of the cells by 50%, compared to the untreated control cells. 

After 24 h of incubation, the activity of DXI-NLC was almost three- and twelve-fold higher than the activity of the empty NLC (DXI was not assessed due to its low water solubility) toward PC-3 and MDA-MB-468, respectively. During this time, the activity of the encapsulated DXI was getting lower; as listed in Table 7, DXI-NLC exhibited much lower activity after 72 h than after 24 h. This is in correlation with the results presented on the release profile, since after 24 h it is predicted that around 90% of the encapsulated DXI will be released. This resulted in a higher antiproliferative activity of DXI-NLC after 24 h than after 48 or 72 h. Interestingly, empty NLCs also pose antitumoral properties by themselves, probably due to the beeswax effect. In this sense, several studies claim that honey-derived products have the ability to kill cancer cell lines [55,56]. Therefore, the combined properties of NLC and DXI have been demonstrated in vitro.

## 3. Materials and Methods

### 3.1. Chemicals and Reagents

S-(+)-ibuprofen (dexibuprofen/DXI) was purchased from Amadis Chemical^®^ (Hangzhou, Zhejiang, China). Beeswax, Tween^®^ 80 (polysorbate 80) and ortho-phosphoric acid (85%) were purchased from Sigma-Aldrich (Madrid, Spain). Miglyol 812 (caprylic/capric triglycerides) was purchased from Roig Farma SA (Terrassa, Spain). Sodium citrate tri-base and urea were purchased from Montplet & Esteban SA (Barcelona, Spain). Methanol (99.8%) was a product of Fisher Scientific (Geel, Belgium). All other reagents including ethanol (99.8%, PanReac AppliChem, Barcelona, Spain) were of analytical grade. Millipore^®^ Milli^®^ Q system (Darmstadt, Germany) purified water was used across all experiments and formulations. 

### 3.2. Methods

#### 3.2.1. Preparation of Nanostructured Lipid Carriers 

DXI-NLC was produced using the high-shear hot homogenization technique [57]. In brief, the lipid phase was prepared by melting DXI, beeswax and Miglyol 812. The aqueous phase was prepared by dissolving Tween 80. After a 30 min water bath (∼90 °C), both phases were mixed and the pre-emulsion was homogenized with an IKA^®^ T10 basic ULTRA-TURRAX^®^ (Staufen, Germany) at a speed of 8000 rpm for 30 s. Subsequently, the mixture was exposed to high-pressure homogenization (HPH) at a pressure of 800 mbar and 85 °C for 3 homogenization cycles using a Stansted pressure cell homogenizer FPG12800 (Stansted Fluid Power, Harlow, UK). The prepared DXI-NLC samples were allowed to settle down for 12 h at room temperature before further experiments. Empty NLCs were prepared using the same procedure without adding DXI.

#### 3.2.2. Physicochemical Characterization of Nanostructured Lipid Carriers

Z_ave_ and PDI were analyzed using dynamic light scattering (DLS) [39]. NLCs were diluted in water (1:10) and Z_ave_ and PDI values were measured using Zetasizer Nano ZS (Malvern, UK). ZP values of DXI-NLC were measured with an electrophoretic light scattering (ELS) technique using Zetasizer Nano ZS (Malvern, UK) equipped with an electrophoresis laser Doppler [39]. NLCs were diluted in water (1:20) and loaded in a folded capillary cell (Malvern, UK) for ZP measurements. All measurements were carried out in triplicate at 25 °C and values are presented as mean ± standard deviation [44,45].

The EE of DXI in DXI-NLC was determined indirectly by measuring the free DXI in the aqueous phase (Equation (1)). Prepared NLCs were diluted in water (1:10) and filtered by centrifugation through Amicon^®^ 0.5 ml Ultracel 100 K filters (Merck KGaA, Darmstadt, Germany) for 15 min at 14,000 rpm to separate the free DXI from the NLCs. Free DXI in the aqueous phase was quantified by reverse-phase high-performance liquid chromatography (RP-HPLC) [39]. In the HPLC system, samples were analyzed using a Waters™ 600 Controller (Waters, Milford, MA, USA), Waters™ 717 plus autosampler (Waters, Milford, MA, USA), and a Kromasil^®^ Classic C18 (5 µm × 4.6 × 150 mm) column (Nouryon, Amsterdam, The Netherlands). The mobile phase constituted an aqueous phase containing 0.05% ortho-phosphoric acid in water and an organic phase containing methanol in a water: methanol ratio of 20:80, and at a 1 mL/min flow rate. A Waters™ 2996 Photodiode array detector (Waters, Milford, MA, USA) was used at 220 nm wavelength [58,59]. Data were processed using Empower^®^ version 3 (Waters, Milford, MA, USA). The calibration curve was calculated using the DXI concentration range between 0.25 and 500 µg/mL against the area of the corresponding detected peaks.

(1) $$\mathrm{EE}\;\left(\%\right)=\frac{\mathrm{total}\;\mathrm{amount}\;\mathrm{of}\;\mathrm{DXI}-\mathrm{free}\;\mathrm{DXI}}{\mathrm{total}\;\mathrm{amount}\;\mathrm{of}\;\mathrm{DXI}}\times 100$$

#### 3.2.3. Optimization of Nanostructured Lipid Carrier Parameters

The optimization of the formulation was carried out by studying the influence of the independent variables (DXI, beeswax, Miglyol 812, polysorbate 80) on the dependent variables (Z_ave_, PDI, ZP and EE) [32]. A two-level factorial design (2^4^) was carried out using Statgraphics 18. The matrix of the design used and the levels of the independent parameters are given in Table 8. 

#### 3.2.4. Interaction Studies and Nanosphere Characterization 

##### Differential Scanning Calorimetry

Thermograms of DXI-NLC, DXI and empty NLCs were obtained using a DSC system (Mettler TA 4000 system, Mettler, Greifensee, Switzerland) equipped with a DSC-25 cell. Dried samples were transferred to perforated aluminum pans and weighed on Mettler M3 Microbalance (Mettler, Greifensee, Switzerland). A pan with indium (purity ≥99.95%, Fluka, Switzerland) was used for the calibration of the calorimetric system. An empty identical aluminum pan was used as a reference. Samples were melted under nitrogen flow at 10 °C/min to measure the melting transition point by heating the ramp from 25 to 100 °C [39]. 

##### Fourier-Transform Infrared Spectroscopy 

The Fourier-transform infrared (FTIR) spectra of DXI-NLC, DXI, and the empty NLC were analyzed. The scan was performed between the range of 525 and 4000 cm^−1^ on a Thermo Scientific Nicolet iZ10 spectrometer equipped with an ATR diamond and a DTGS detector [39]. 

##### X-ray Diffraction

X-ray diffraction (XRD) was carried on DXI-NLC, DXI, and the empty NLC. XRD was performed to analyze the amphous or crystalline state of the formulations with and without DXI. Dried formulations and DXI were placed in between the polyester films and exposed to CuK” radiation (45 kV, 40 mA, λ = 1.5418 Å). Measurements were carried at 2θ from 2° to 60° with a step size and time interval of 0.026° and 200 s, respectively [39]. 

##### Transmission Electron Microscopy

Morphological observations of DXI-NLC were carried out under transmission electron microscopy (TEM) on a Jeol 1010 microscope (Akishima, Japan). DXI-NLC were diluted (1:5) with water and placed on a surface of copper grids activated with UV light. Subsequently, negative staining was performed using 2% uranyl acetate [58]. 

#### 3.2.5. In Vitro Drug Release 

In vitro drug release was examined with the direct dialysis technique [60]. The release of DXI from DXI-NLC (*n* = 3) was estimated against the same concentration of DXI (*n* = 3) by placing the samples in a 500 µL dialysis cassette (10K MWCO, Slide-A-LyzerTM, Thermo Scientific, Rockford, IL, USA). The release medium was prepared with sodium citrate and urea to improve the hydrophilicity of DXI in a proportion of DXI:S sodium citrate: urea 1:3:7, as explained elsewhere [41]. The release medium was placed under magnetic stirring and incubated at 37 °C. At various time intervals, samples of 400 µL of release medium were collected and replaced by 400 µL of clean release medium. Collected samples were analyzed with RP-HPLC [39]. The amount of DXI released was plotted as cumulative drug release against time (h) in Graph Pad Prism version 5.0, and fitted to the most common biopharmaceutical equations (Higuchi, Korsmeyer–Peppas, hyperbola and first-order equation).

#### 3.2.6. Short-Term Stability of Solid Lipid Nanoparticles 

Short-term stability was assessed by preparing DXI-NLC samples and storing them at three different temperatures (4, 25 and 37 °C). The physicochemical parameters (Z_ave_, PDI, ZP and EE) were analyzed monthly for 60 days.

#### 3.2.7. Sterilization by Gamma Radiation 

Gama radiation was carried out in order to sterilize the samples to develop further cellular assays. DXI-NLC samples were sterilized using a dose of 25 KGy of 60 Co as the gamma irradiation source (Aragogamma, Barcelona, Spain) [44]. Physicochemical parameters were measured before and after gamma radiation.

#### 3.2.8. In Vitro Cytotoxicity Assay

##### Cell Lines

Human prostate cancer PC-3 cell line was obtained from the European Collection of Authenticated Cell Cultures (UK); human breast cancer MDA-MB-468 cell line cells were obtained from the Leibniz Institute DSMZ—German Collection of Microorganisms and Cell Cultures (Germany). All the cell lines were maintained at the Hirszfeld Institute of Immunology and Experimental Therapy, PAS, Wroclaw, Poland. PC-3 and MDA-MB-468 cells were cultured in RPMI 1640 +GlutaMAX medium (Gibco, UK) with 10% (PC-3) or 20% (MDA-MB-468) fetal bovine serum (FBS) (HyClone, Cytiva). All culture media were supplemented with 100 units/mL penicillin, (Polfa Tarchomin S.A., Poland) and 100 µg/mL streptomycin (Merck, Germany). All cell lines were grown at 37 °C with a 5% CO_2_ humidified atmosphere.

##### Determination of Antiproliferative Activity

DXI-NLC and the empty NLCs were assayed between 0.8 and 100 µM. Formulations were diluted based on the total encapsulated DXI concentration (1.49 mg/ml) in DXI-NLC. Then, 24 h before adding NLCs, the cells were placed in 96-well plates (Sarstedt, Germany) at a density of 1 × 10^4^ cells per well. The antiproliferative activity assay was performed after 24, 48 and 72 h of exposure to NLCs. The in vitro cytotoxic effect was examined using the SRB assay, as previously described [48]. The results were calculated as an IC_50_: the concentration of NLCs which was cytotoxic for 50% of the cancer cells. IC_50_ values were calculated for each experiment separately using the Prolab-3 system based on Cheburator 0.4 software [49], and mean values ± SD are presented in Table 4. Each compound in each concentration was tested in triplicate in a single experiment which was repeated 3–5 times.

#### 3.2.9. Statistics 

Student’s t-tests were carried for two-group comparations to explain the significant differences between treatments using Statgraphics 18. A statistically significant difference was considered at *p* ≤ 0.05. 

## 4. Conclusions

In the present study, a novel drug delivery system based on nanostructured lipid carriers encapsulating DXI has been developed and optimized for the treatment of tumoral diseases. DXI-NLC were prepared using the high-shear homogenization procedure using HPH, which offers an easy industrial scale-up process. Moreover, DXI-NLC was confirmed to be stable for 2 months at 4 and 25 °C and to provide a prolonged drug release, slower than free DXI. Furthermore, DXI-NLC showed suitable physicochemical properties that allow it to act as an effective antitumoral agent, as demonstrated in breast and prostate cancer cell lines. Therefore, DXI-NLC constitutes a promising formulation to be used as antitumoral agent, and is especially effective against breast cancer cells.

## Figures and Tables

**Table 1:** Concentrations corresponding to the preformulation studies of NLCs encapsulating DXI.

Compounds	Concentrations of Selected Excipients	
	Preformulation A	Preformulation B
DXI (mg/mL)	1	1
Beeswax (%)	4	4
Castor oil (%)	2	0
Miglyol 812 (%)	0	2
Tween 80 (%)	3	3

**Table 2:** Physicochemical characterization of the synthesis of nonoptimized DXI-NLCs. (A) NLCs with beeswax and castor oil; (B) NLCs with beeswax and Miglyol 812.

Preformulation	Z_ave_ (nm)	PDI	ZP (mV)	EE (%)
A	129.8 ± 1.3	0.232 ± 0.025	−18.9 ± 0.5	96.94
	123.1 ± 0.6	0.223 ± 0.021	−17.7 ± 0.3	96.89
B	103.8 ± 1.1	0.171 ± 0.019	−15.9 ± 0.2	97.02
	92.35 ± 2.32	0.188 ± 0.012	−14.8 ± 0.3	95.69

Results are presented as mean ± SD (*n* = 3); Z_ave_: mean average size; PDI: polydispersity index; ZP: zeta potential; EE: encapsulation efficiency.

**Table 3:** Values of the experimental factors according to the matrix designed by 2^4^ + star central composite rotatable factorial design parameters and measured responses.

	DXI		Beeswax		Miglyol 812		Tween 80		Parameters			
	Coded Level	mg/mL	Coded Level	%	Coded Level	%	Coded Level	%	Z_ave_ (nm)	PDI	ZP (mV)	EE (%)
**Factorial Points**												
F1	1	1.25	1	5	1	2.5	1	4	89.1 ± 1.09	0.17 ± 0.01	−17.6 ± 2.91	98.96
F2	−1	0.75	1	5	1	2.5	1	4	103.9 ± 1.96	0.18 ± 0.03	−16.5 ± 0.61	97.72
F3	1	1.25	−1	3	1	2.5	1	4	72.52 ± 0.84	0.18 ± 0.01	−14.9 ± 2.01	98.87
F4	−1	0.75	−1	3	1	2.5	1	4	77.43 ± 1.46	0.2 ± 0.01	−15.6 ± 1.57	96.01
F5	1	1.25	1	5	−1	1.5	1	4	95.51 ± 0.06	0.19 ± 0.01	−16.7 ± 1.65	97.14
F6	−1	0.75	1	5	−1	1.5	1	4	98.25 ± 1.93	0.19 ± 0.05	−18.7 ± 0.82	96.52
F7	1	1.25	−1	3	−1	1.5	1	4	71.73 ± 0.59	0.13 ± 0.01	−13.1 ± 2.46	97.91
F8	−1	0.75	−1	3	−1	1.5	1	4	70.25 ± 0.72	0.22 ± 0.01	−16.7 ± 0.7	95.7
F9	−1	0.75	1	5	1	2.5	−1	2	154.3 ± 2.44	0.15 ± 0.04	−19.5 ± 0.51	98.96
F10	−1	0.75	−1	3	1	2.5	−1	2	126.9 ± 1.3	0.17 ± 0.01	−20 ± 2.01	99.11
F11	−1	0.75	1	5	−1	1.5	−1	2	145.8 ± 0.58	0.12 ± 0.03	−20.6 ± 0.4	99.48
F12	−1	0.75	−1	3	−1	1.5	−1	2	112.6 ± 1.23	0.18 ± 0.01	−17.8 ± 1.47	96.28
F13	1	1.25	1	5	1	2.5	−1	2	160.4 ± 1.43	0.12 ± 0.02	−21.8 ± 2.66	96.09
F14	1	1.25	−1	3	1	2.5	−1	2	129.7 ± 2.35	0.15 ± 0.01	−18.1 ± 0.54	98.12
F15	1	1.25	1	5	−1	1.5	−1	2	146.9 ± 0.98	0.09 ± 0.04	−20.6 ± 0.76	99.88
F16	1	1.25	−1	3	−1	1.5	−1	2	112.7 ± 0.78	0.17 ± 0.03	−18.1 ± 0.7	97.46
**Axial Points**												
F17	−2	0.5	0	4	0	2	0	3	108.70 ± 1.82	0.17 ± 0.01	−21.7 ± 1.48	99.29
F18	2	1.5	0	4	0	2	0	3	106.7 ± 0.89	0.17 ± 0.02	−15.9 ± 0.53	97.87
F19	0	1	0	4	0	2	2	5	78.94 ± 1.56	0.18 ± 0.02	−14.1 ± 0.51	96.3
F20	0	1	0	4	2	3	0	3	116.7 ± 1.01	0.18 ± 0.01	−17.7 ± 0.38	98.52
F21	0	1	0	4	−2	1	0	3	100 ± 3.07	0.2 ± 0.03	−19.4 ± 0.06	97.65
F22	0	1	2	6	0	2	0	3	112.1 ± 2.12	0.17 ± 0.02	−20.3 ± 0.38	98.89
F23	0	1	−2	2	0	2	0	3	71.86 ± 0.41	0.16 ± 0.08	−16.8 ± 0.22	98.37
F24	0	1	0	4	0	2	−2	1	182.8 ± 0.27	0.16 ± 0.02	−20.8 ± 0.27	98.54
**Central Points**												
F25	0	1	0	4	0	2	0	3	110.4 ± 2.03	0.16 ± 0.03	−15.6 ± 0.31	97.2
F26	0	1	0	4	0	2	0	3	91.75 ± 1.77	0.18 ± 0.01	−17 ± 0.72	98.3

Results are presented as mean ± SD (*n* = 3); F1-F26: formulations of DXI-NLCs; Z_ave_: mean average size; PDI: polydispersity index; ZP: zeta potential; EE: encapsulation efficiency.

**Table 4:** Predicted and expected physiochemical parameters and EE of DXI-NLC.

Parameters	Predicted Values	Experimental Values
Z_ave_ (nm)	~150	152.3 ± 1.6
PDI	<0.18	0.149 ± 0.03
ZP (mV)	−20	−19.8 ± 0.764
EE (%)	>95	99.17

Experimental values are presented as mean ± SD (*n* = 3); Z-Ave: mean size; PDI: polydispersity index; ZP: zeta potential; EE (%): encapsulation efficacy.

**Table 5:** Results of hyperbola equation for cumulative DXI release vs. time.

Parameters	DXI	DXI-NLC
B_max_ ± SD (%)	118.30 ± 5.75	91.09 ± 3.14
K_d_ ± SD (h)	2.24 ± 0.28	2.90 ± 0.22
R^2^ value	0.9264	0.9763

B_max_: maximum binding capacity; K_d_: equilibrium dissociation constant.

**Table 6:** Results of stability studies of DXI-loaded nanostructured lipid carriers (NLCs).

Day	T (°C)	Z_ave_ (nm)	PDI	ZP (mV)
0	4	152.3 ± 1.6	0.149 ± 0.03	−19.8 ± 0.76
	25	152.3 ± 1.6	0.149 ± 0.03	−19.8 ± 0.76
	37	152.3 ± 1.6	0.149 ± 0.03	−19.8 ± 0.76
30	4	152.6 ± 2.1	0.145 ± 0.01	−19.2 ± 0.53
	25	150.7 ± 2.4	0.143 ± 0.02	−19.6 ± 0.4
	37	152.4 ± 2.9	0.143 ± 0.02	−18 ± 0.17
60	4	149.8 ± 0.8	0.158 ± 0.01	−22.5 ± 0.15 *
	25	150.9 ± 1.9	0.140 ± 0.04	−21.6 ± 0.17 *
	37	150.4 ± 1	0.158 ± 0.01	−17.4 ± 0.4 *

Results are presented as mean ± SD (*n* = 3); T (°C): temperature; Zave: mean size; PDI: polydispersity index; ZP: zeta potential; * significant difference.

**Table 7:** IC_50_ values of DXI-NLC and empty NLC at several incubation times.

Formulation	IC_50_ (µM)					
	PC-3			MDA-MB-468		
	24h	48h	72h	24h	48h	72h
**Empty NLC**	28.5 ± 4.3	14.9 ± 5.6	12.9 ± 1.3	42.5 ± 11.8	69.6 ± 27.5	11.6 ± 6.7
**DXI-NLC**	10.1 ± 3.1	72.9 ± 21.3	60.8 ± 10.3	3.4 ± 0.4	82.4 ± 65.2	66.1 ± 22.4

Results are presented as mean ± SD (*n* = 3); IC_50_: 50% inhibitory concentration; PC-3: human prostate cancer cell line; MDA-MB-468: human breast cancer cell line 3.

**Table 8:** Chemical concentrations employed for optimization.

Compounds	Levels				
	−2	−1	0	1	2
DXI (mg/mL)	0.5	0.75	1	1.25	1.5
Beeswax (%)	2	3	4	5	6
Miglyol (%)	1	1.5	2	2.5	3
Tween 80 (%)	1	2	3	4	5
